# Techno-economic and resource analysis of hydroprocessed renewable jet fuel

**DOI:** 10.1186/s13068-017-0945-3

**Published:** 2017-11-09

**Authors:** Ling Tao, Anelia Milbrandt, Yanan Zhang, Wei-Cheng Wang

**Affiliations:** National Renewable Energy Laboratory, 15013 Denver West Parkway, Golden, CO 80401 USA

**Keywords:** Techno-economics analysis, Feedstock, Hydroprocessed renewable jet fuel, Alternative jet fuel, Resources, Lipids

## Abstract

**Background:**

Biomass-derived jet fuel is an alternative jet fuel (AJF) showing promise of reducing the dependence on fossil fuel and greenhouse gas emissions. Hydroprocessed esters and fatty acids (HEFA) concept is also known as one of the pathways for producing bio jet fuel. HEFA fuel was approved by the American Society for Testing and Materials in 2011, and can be blended up to 50% with conventional jet fuel. Since then, several HEFA economic and life-cycle assessments have been published in literature. However, there have been limited analyses on feedstock availability, composition, and their impact on hydrocarbon yield (particularly jet blendstock yield) and overall process economics.

**Results:**

This study examines over 20 oil feedstocks, their geographic distribution and production levels, oil yield, prices, and chemical composition. The results of our compositional analysis indicate that most oils contain mainly C_16_ and C_18_ fatty acids except pennycress, yellow grease, and mustard, which contain higher values and thus would require hydrocracking to improve jet fuel production. Coconut oil has a large content of shorter carbon fatty acids, making it a good feedstock candidate for renewable gasoline instead of jet substitutes’ production. Techno-economic analysis (TEA) was performed for five selected oil feedstocks—camelina, pennycress, jatropha, castor bean, and yellow grease—using the HEFA process concept.

**Conclusion:**

The resource analysis indicates that oil crops currently grown in the United States (namely soybean) have relatively low oil yield when compared to oil crops grown in other parts of the world, such as palm, coconut, and jatropha. Also, non-terrestrial oil sources, such as animal fats and greases, have relatively lower prices than terrestrial oil crops. The minimum jet fuel selling price for these five resources ranges between $3.8 and $11.0 per gallon. The results of our TEA and resource studies indicate the key cost drivers for a biorefinery converting oil to jet hydrocarbons are as follows: oil price, conversion plant capacity, fatty acid profile, addition of hydrocracker, and type of hydroprocessing catalysts.

**Electronic supplementary material:**

The online version of this article (10.1186/s13068-017-0945-3) contains supplementary material, which is available to authorized users.

## Background

Aviation fuel has more stringent quality requirements and fuel specifications than fuels used in road transportation. Jet fuel is a type of aviation fuel designed specifically to power gas-turbine engines. According to a report from the United States (US) Energy Information Administration (EIA) [1], about 10% of each barrel (42 gallons per barrel) of crude oil is used to produce jet fuel. The worldwide aviation industry consumes approximately 63–134 billion gallons of conventional jet fuel per year [2, 3]. Based on the 2015 estimates from the EIA, jet fuel consumption in the transportation sector in the US is 23.7 billion gallons, and expenditures for this fuel are $39 billion dollars [4]. Fuel is the largest operating cost in the aviation industry, and the unstable prices of crude oil hamper long-term planning and expense budgeting. Jet fuel from renewable sources such as biomass can reduce the dependency of the aviation industry on one single energy source, potentially reducing the risk of the petroleum prices volatility [5], and potentially reducing greenhouse gas (GHG) emissions [2]. For the US Department of Defense alternative fuel initiatives, the US Air Force has set goals to test and certify all aircrafts and systems on a 50:50 alternative fuel blend and to ensure that 50% of the domestic aviation fuel used by the Air Force comes from an alternative fuel blend by 2025 [6, 7]. The US Navy’s goal is to run ships and aircraft entirely on alternative fuel blends and to achieve 50% of the Navy’s total energy use from alternative sources by 2020 [6].

Technical certification of alternative fuels is primarily led by the American Society for Testing and Materials (ASTM) with support from the Commercial Aviation Alternative Fuels Initiative and the US Air Force. Certain biojet fuels can now be blended up to 50% with conventional commercial and military jet (or aviation turbine) fuel [8, 9]. These include Fischer–Tropsch fuels using solid biomass resources; hydroprocessed esters and fatty acids (HEFA) fuels derived from used cooking oil, animal fats, algae, and vegetable oils; and alcohol-to-jet fuels produced from isobutanol and blended to a maximum level of 30%.

HEFA fuel properties are similar to conventional petroleum fuel, but the fuel has the advantages of a higher cetane number, lower aromatic content, lower sulfur content, and potentially lower GHG emissions [10]. The hydroprocessing conversion technologies (e.g., hydrotreating, deoxygenation, isomerization, and hydrocracking) are at a relatively high maturity level and are commercially available. These processes are commonly used in today’s refineries to produce transportation fuels. Since 2008, many test flights using HEFA fuel from various oil-based feedstocks (e.g. jatropha, algae, camelina, and yellow grease) have been conducted by military and commercial entities [11–19]. Neste Oil and Honeywell Universal Oil Products (UOP) are one of the leading companies producing HEFA fuel for the aviation biofuels market [20–23].

There are a few economic analyses of HEFA fuel in literature [8, 24]. While there is some information on feedstock availability and composition, there is a general lack of understanding of their impact on hydrocarbon yield (particularly jet blendstock yield) and overall process economics. The goal of this study is to improve the understanding of HEFA fuel economics and thus support future development of this technology. To achieve this goal, we defined three objectives: (1) conduct a resource assessment that evaluates the geographic distribution and production levels of major oil sources, their oil yield, and prices; (2) analyze the chemical composition of oil feedstock, namely their free fatty acid (FFA) profile; and (3) conduct a comprehensive but comparative techno-economic analysis (TEA) on five selective oil feedstocks. The market will ultimately decide which resources would be used for what purposes. Our paper only states the possibilities and serves as a reference if these feedstocks are used for biofuels production. TEA is an essential and powerful tool used to understand economic potential of a technology strategy, effectively prioritize research directions, and suggest new research toward an economically viable process strategy.

## Methods

### Resource analysis

We examined over 20 sources for HEFA production as summarized in Table 1. Our primary focus was on sources applicable to the US, although some additional feedstocks were included due to their import in the country, importance in the international oilseed market, or receiving global attention as an emerging biofuel feedstock. Price and yield data for these sources were gathered and analyzed. Data providers included the US Department of Agriculture (USDA), consulting agencies, and private companies engaged in feedstock production or distribution. For most feedstocks, the 2014 annual average price was obtained. For feedstocks with a missing of 2014 price information, we used the most recent data at a given point in time (within the 2012–2013 timeframe) or model-derived estimates. Information on the average yield for the reviewed oil crops was also gathered. We recognized that crop yields vary under different agro-climatic conditions but for the purpose of this study, we assumed that the average value was a reasonable proxy for the midpoint of a yield range. We were unable to conduct sensitivity analyses with low and high yield at this time. In addition to these activities, we gathered data on production of the major oil crops in the US and a map was generated to illustrate the geographic distribution of these resources by county.

**Table 1:** Sources for hydroprocessed renewable jet fuel

Vegetable oil	
Palm/Palm kernel	*Elaeis guineensis*
Coconut	*Cocos nucifera*
Jatropha	*Jatropha curcas*
Castor	*Ricinus communis*
Rapeseed	*Brassica napus*
Canola	*Brassica napus*, cultivar
Pennycress	*Thlaspi arvense*
Peanut (groundnut)	*Arachis hypogaea*
Sunflower	*Helianthus annuus*
Safflower	*Carthamus tinctorius*
Camelina	*Camelina sativa*
Mustard	*Brassica juncea*
Linseed (flax)	*Linum usitatissimum*
Soybean	*Glycine max*
Cottonseed	*Gossypium hirsutum*
Corn	*Zea mays*
Animal fats	
Lard	Edible pork fat, rendered and unrendered
Choice white grease	Inedible pork fat derived primarily from pork tissue
Edible tallow	Beef fat suitable for human consumption
Inedible tallow	Beef fat unsuitable for human consumption
Poultry fat	Fat obtained from chicken rendering and processing
Grease	
Yellow grease	Derived from used cooking oil generated by commercial and industrial cooking operations. It may also contain rendered animal fat
Brown grease	Waste grease recovered from traps installed in the sewage lines of restaurants/food processing plants and wastewater treatment plants.
Aquatic microorganisms	
Algae	A large group of simple plant-like photosynthetic organisms

Five oil sources were selected for the TEA: camelina, pennycress, jatropha, castor bean, and yellow grease. The five sources were selected for the following reasons: non-food feedstocks (pennycress and castor bean), promising for the US agro-climatic conditions (camelina, pennycress, and castor bean), low cost and readily available (yellow grease), receiving global attention (jatropha), and high yield among terrestrial plants (jatropha and castor bean). Additionally, some of these sources were less studied as potential jet fuel feedstock (e.g., pennycress and castor bean), thus we saw an opportunity for this study to improve the knowledge base for these feedstocks. Moreover, alternative jet fuel (AJF) produced from camelina oil, jatropha oil, and yellow grease has been tested in aircrafts, which indicated market interest in these sources [24]. Algae was also considered a promising biofuel feedstock but it was not included in our analysis because there have been many other studies on algae productivity and economics over the years [25–31]. Below is a brief description of the five selected oil sources.

Camelina is an annual flowering plant (commonly known as gold-of-pleasure or false flax) of the *Brassicaceae* family that includes the well-known oil crops rapeseed, canola, and mustard. Camelina has a high oil content (about 35% oil) and improved drought tolerance and water use efficiency (yield vs. evapotranspiration) when compared to other oilseed crops [32]. These characteristics make camelina a suitable biofuel crop for the arid western states, an area generally lacking opportunities for growing biofuel feedstock. Camelina production requires low agricultural input and the same equipment as wheat and thus fits well into a dryland crop rotation; it could replace fallow, provide an energy crop, and would not compete with food crops production [33]. Because camelina oil is high in omega-3 fatty acids, perceived to have health benefits, it is considered high-quality edible oil. This may lead to feedstock competition between the biofuels and the food industries as well as high feedstock prices.

Pennycress, also known as stinkweed or French-weed, is a winter annual belonging to the *Brassicaceae* family. It has been growing as a weed in the Midwest but there have been efforts to cultivate it in recent years. The plant has potential to serve in a summer/winter rotational cycle with conventional commodity crops (such as corn or soybean), thus not displacing existing agricultural production [34]. Field pennycress is tolerant of fallow lands, requires minimal agricultural inputs (fertilizer, pesticides, water), it is a non-food crop, it is compatible with existing farm infrastructure, and has high oil content (up to 36% oil) [34]. The plant has been researched by the USDA and other organizations such as the plant science startup Arvegenix, a leading developer of field pennycress, focused on the genetic improvement and commercialization of the plant.

Jatropha is a tropical perennial shrub that has received a lot of attention in recent years. This multipurpose plant is already used as a live fence and to control erosion; the oil extracted from the seeds (about 35% or more) is used for medicinal purposes and soap making; and the seedcake is used as organic fertilizer and animal feed [35]. Some 10 years ago, the plant’s oil was targeted as feedstock for biofuels production or a direct substitute for petroleum diesel in power generators. Jatropha was promoted as a drought-resistant, low-input plant, able to deliver high-quality biofuel on marginal lands [36]. Labeled as a “miracle crop” [37–39], the plant attracted large investments. However, jatropha lost its appeal during the recession as farmers realized that the yield is far lower than predicted. Jatropha may hold potential for biofuels production but there are many uncertainties surrounding its cultivation; primarily because while it grows abundantly in the wild, it has never been domesticated. Recently, SGB, an agricultural biotechnology company, claimed to have succeeded in domesticating the plant through advances in molecular genetics and DNA sequencing technology, a process that once took decades [40].

Yellow grease is essentially rendered used cooking oil (restaurant grease) that meets the following specifications: FFA maximum of 15% and moisture, impurities, and unsaponifiables of less than 2 with 1% maximum water [41]. Yellow grease is a commodity in the US and has recently become increasingly valuable since it is now used for production of biofuels. Historically, it has been used as an animal feed additive, for production of plastics, textiles, and cosmetics, in soap making, and as a lubricant. Yellow grease is an attractive feedstock for the biofuels industry because it is readily available and relatively inexpensive.

Castor bean is a perennial plant in tropical and subtropical regions and can be grown as an annual in colder climates. Castor oil is essential to the chemical industry because it is the only commercial source of hydroxylated fatty acids (HFA)—ricinoleic acid (C18:1-OH). It is used in paints, coatings, inks, lubricants, and a wide variety of other products [42]. Due to a combination of economic factors, allergenic reactions associated with growing and processing the plant, and toxicity of the seed meal (the seeds contain ricin, a toxic protein), production in the United States ceased in the early 1970s, and currently the industry depends on imports, primarily from India. Despite the controversy surrounding its production, there is a growing interest in domestic castor production because of reported high oil yield and suitability on marginal lands. Researchers at Texas AgriLife Extension reported oil yield at about 50% and found castor to be drought and salt tolerant, therefore a suitable oil crop for select areas of Texas and potentially the whole Southwest [43]. Researchers at the University of California—Davis are also testing castor as a potential feedstock for biofuels production [43]. Efforts to reduce toxicity and make the plant safe are underway at Texas Tech University and Mississippi State University [43, 44].

There are other potential oil crops for HEFA including Lesquerella (*Lesquerella fendleri*), Cuphea (*Cuphea* spp., *C. Viscosissima*), and Crambe (*Crambe abyssinica*). Lesquerella, commonly known as bladderpod, is a native plant to the southwestern United States and Mexico. This crop is desirable due to the high level of HFA in the oil, lesquerolic acid (C20:1-OH), similar to that in castor oil but without the toxic ricin. Thus, it could be a safer alternative to the imported castor oil. Similar to castor, lesquerella methyl esters have been shown to increase lubricity in ultra-low sulfur diesel at concentrations as low as 0.25% [45]. Cuphea (also known as blue waxweed, clammy cuphea, or tarweed) is a plant native to the Americas, adapted to the temperate regions. The plant species offers high levels of medium-chain fatty acids (C_8_–C_12_) used in the production of lubricants, soaps, detergents, cosmetics, and personal-care products, and is currently supplied in the US by imported coconut and palm oil [46]. Therefore, the plant offers a domestic alternative to these tropical sources and a business opportunity for farmers in the temperate climate for no other temperate oilseed crop has been found to provide these lipids [46]. Moreover, cuphea oil is reported to have low viscosity, which makes it suitable for direct use as fuel—petroleum diesel blends with cuphea oil performed well in engine durability tests [46]. Crambe, also known as Abyssinian kale, is believed to be of Mediterranean origin and has been grown in a wide range of climatic conditions [47]. There has been limited production in the United States, mostly in North Dakota, since 1990 [48]. The seed oil of crambe is non-edible and contains a high level of erucic acid, an important feedstock for the oleo-chemical industry. Crambe is reported to have high yield potential, resistance to insect feeding (possibly due to high glucosinolate content), and more tolerance than canola to abiotic stress such as salinity, cold temperature, heat and drought, and heavy metal exposure [47]. These less-known oil crops were not included in the TEA.

### Process design

**Fig. 1:**
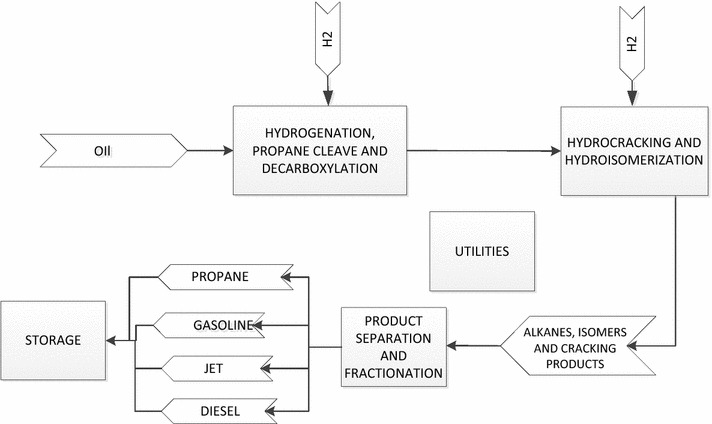
Schematic process flow diagram

Although feedstocks for HEFA processes include natural oils derived from plants, animal fats, post-consumer wastes (e.g., yellow grease), and aquatic microorganisms such as algae and cyanobacteria, the generic process concept is very similar. A representative process flow diagram is shown in Fig. 1, including processes of hydrogenation, propane cleave, hydrocracking and hydroisomerization, and product fractionation.

Bio-oils are sent to the hydroprocessing facility (first block in Fig. 1), fundamentally with three reaction steps—hydrogenation, propane cleave, and decarboxylation—according to patents by UOP and Syntroleum [49, 50]. First, catalytic hydrogenation could be used to convert liquid-phase unsaturated FFAs or glycerides into saturated with the addition of hydrogen (H_2_) [51]. Hydrogenation takes place to saturate the double bonds in the unsaturated triglycerides [52]. The reaction equations are [52]:

1 $${\text{triolein}} + 3 {\text{H}}_{2} \to {\text{tristearin}}$$

2 $${\text{trilinolein}} + 6 {\text{H}}_{2} \to {\text{tristearin}}$$

3 $${\text{trilinolenin}} + 9 {\text{H}}_{2} \to {\text{tristearin}}$$

The second step is to cleave the propane and produce three moles of FFAs [10] per mole of triglycerides. The glycerol portion of the triglyceride molecule is converted into propane by adding H_2_. The propane cleave process removes the propane backbone from the molecule, turning glycerides into three fatty acids, shown in Eqs. 4–9:

4 $${\text{trimyristin}} + 3 {\text{H}}_{2} \to 3 {\text{C}}_{14} {\text{H}}_{27} {\text{COOH}} + {\text{C}}_{3} {\text{H}}_{8}$$

5 $${\text{tripalmitin}} + 3 {\text{H}}_{2} \to 3 {\text{C}}_{16} {\text{H}}_{31} {\text{COOH}} + {\text{C}}_{3} {\text{H}}_{8}$$

6 $${\text{tristearin}} + 3 {\text{H}}_{2} \to 3 {\text{C}}_{18} {\text{H}}_{35} {\text{COOH}} + {\text{C}}_{3} {\text{H}}_{8}$$

7 $${\text{triarachidin}} + 3 {\text{H}}_{2} \to 3 {\text{C}}_{20} {\text{H}}_{39} {\text{COOH}} + {\text{C}}_{3} {\text{H}}_{8}$$

8 $${\text{tribehenin}} + 3 {\text{H}}_{2} \to 3 {\text{C}}_{22} {\text{H}}_{43} {\text{COOH}} + {\text{C}}_{3} {\text{H}}_{8}$$

9 $${\text{trilignocerin}} + 3 {\text{H}}_{2} \to 3 {\text{C}}_{24} {\text{H}}_{47} {\text{COOH}} + {\text{C}}_{3} {\text{H}}_{8}$$

The third reaction is to remove the oxygen from the fatty acids [53]. There are three pathways occurring in this stage: decarboxylation, decarbonylation, and hydrodeoxygenation. The decarboxylation pathway removes oxygen in the form of carbon dioxide (CO_2_), decarbonylation removes oxygen in the form of carbon monoxide (CO), and hydrodeoxygenation removes oxygen in the form of H_2_O. Decarboxylation is chosen in this study, using Eqs. 10–15, while other mixed decarboxylation and hydrodeoxygenation are studied in the sensitivity analysis.

10 $${\text{C}}_{14} {\text{H}}_{27} {\text{COOH}} \to {\text{C}}_{13} {\text{H}}_{28} + {\text{CO}}_{2}$$

11 $${\text{C}}_{16} {\text{H}}_{31} {\text{COOH}} \to {\text{C}}_{15} {\text{H}}_{32} + {\text{CO}}_{2}$$

12 $${\text{C}}_{18} {\text{H}}_{35} {\text{COOH}} \to {\text{C}}_{17} {\text{H}}_{36} + {\text{CO}}_{2}$$

13 $${\text{C}}_{20} {\text{H}}_{39} {\text{COOH}} \to {\text{C}}_{19} {\text{H}}_{40} + {\text{CO}}_{2}$$

14 $${\text{C}}_{22} {\text{H}}_{43} {\text{COOH}} \to {\text{C}}_{21} {\text{H}}_{44} + {\text{CO}}_{2}$$

15 $${\text{C}}_{24} {\text{H}}_{47} {\text{COOH}} \to {\text{C}}_{23} {\text{H}}_{48} + {\text{CO}}_{2}$$

The reaction temperature and pressure for the combined step of hydrogenation, propane cleave, and decarboxylation, are 400 °C and 9.2 megapascal (resulting in the total conversion of 91.9% [52, 54]. The catalyst used in this process is Pd/γ-Al2O3 and the catalyst-to-oil ratio is 0.088. The H_2_ gas is fed into the reactor for the hydrogenation and propane cleave. The H_2_ usage is calculated based on the H_2_ required for saturating the double bonds of the unsaturated triglycerides and cleaving the propane from the glycerol backbone [52, 53]. For instance, for every mole of triolein, trilinolein, and trilinolenin, 3, 6, and 9 mol of (H_2_) would be required, respectively. In addition, for removing the propane molecule from the triglycerides, 3 mol of H_2_ are required [52, 53] per mole of triglycerides. The resulting products contain liquid hydrocarbons and gas products, including CO_2_, H_2_ and propane. The gas is purged and is sent to a vapor–liquid separator to remove the gas phase products. The liquid portion is routed to the second block (shown in Fig. 1). The second hydrotreating step includes hydrocracking and hydroisomerization reactions. To meet the jet fuel specification, the produced AJF has to have not only a high flash point, but also good cold flow properties. Therefore, with the addition of a processing step of hydrocracking and hydroisomerization, the normal paraffins produced from deoxygenation convert to a synthetic paraffinic kerosene (SPK) product [51]. The cracking and isomerization reactions are either concurrent or sequential [51]. Studies have shown that isomerization of straight-chain alkanes occur first and cracking is a sequential reaction. The isomerization process takes the straight-chain hydrocarbons and turns them into the branched structures to reduce the freeze point to meet the jet fuel standard [55]. It is accompanied by a hydrocracking reaction, which results in minimum yield loss from the isomerized species. Sometimes the hydroisomerization will accompany cracking, which reduces the chain length and produces more molecules. The hydroisomerization/cracking reaction is operated at a temperature of 355 °C, pressure of 600 lb per square inch gage, an liquid hourly space velocity of 1(h^−1^), and a H_2_/feed ratio of 50 standard cubic feet/gal [50, 56]. The catalyst can be selected as Pt/HZSM-22/γ-Al2O3 [52]. The product distribution and mass yield are based on Abhari’s work [50]. In this case, large molecules are assumed to crack into small ones and then become partially isomerized, as shown in Eq. 16.

16 $$n - {\text{alkanes }} \to n - {\text{alkanes}} + {\text{isomers}}$$

Bifunctional catalysts containing metallic sites for hydrogenation/dehydrogenation and acid sites for selective isomerization via carbenium ions could be used in isomerization [57]. In a typical isomerization reaction, normal paraffins are dehydrogenated on the metal sites of the catalyst and react on the acid sites to produce olefins protonate with formation of the alkylcarbenium ion. The alkylcarbenium ion is rearranged to monobranched, dibranched, and tribranched alkylcarbenium ions on the acid site. The branched alkylcarbenium ions are deprotonated and hydrogenated to produce the corresponding paraffins [58]. The choice of catalyst will result in variation of cracking at the end of the paraffin molecule and, therefore, adjust the yield of jet blendstocks [51]. This study assumed that the catalyst is used with a weight hourly space velocity (WHSV) of 2 h^−1^, and is replaced every half year.

The hydroisomerization and hydrocracking processes are followed by a fractionation process to separate the mixtures to paraffinic kerosene, paraffinic diesel, naphtha, and light gases. The hydrocracking reactions are exothermic and result in the production of lighter liquids and gas products. They are relatively slow reactions; thus, most of the hydrocracking takes place in the last section of the reactor. The hydrocracking reactions primarily involve cracking and saturation of paraffins. Over-cracking will result in low yields of jet-fuel-range alkanes and high yields of light species ranging from C_1_ to C_4_, and naphtha ranging from C_5_ to C_8_. The bi-functional catalysts used for isomerization contain platinum-containing zeolite catalysts at 1 h^−1^ WHSV in the 250 °C fixed bed reactor similar to the hydrotreating step. Hydroisomerization catalyst life is assumed 5 years, and an atmosphere of H_2_ is used to minimize carbon deposits on the catalyst but H_2_ consumption is negligible.

In the TEA model, C_15_–C_23_ compounds are modeled to be hydrocracked completely to a mixture of hydrocarbons. For instance, if the compound is C_15_, the mixture of hydrocarbons ranges from CH_4_ to C_14_. Both of these are not ideal jet fuel range hydrocarbons and also potentially have lower economic value than diesel or jet fuel.

#### Product separation and fractionation

Unlike biodiesel production through transesterification, HEFA biofuel production requires H_2_ to hydrotreat the biomass. It is suggested that the capital cost for HEFA is 20% higher than that of biodiesel production due to the hydrotreating process [59] if compared with the transesterification process. However, the co-products from HEFA—naphtha, liquefied petroleum gas (LPG), propane, and diesel—have more credits [59]. The hydrocarbon products from the hydroisomerization/cracking reactor are sent to the first distillation column to remove gaseous products. The gaseous products, which contain propane, H_2_, CO_2_, and trace amounts of liquid hydrocarbons, are subjected to further separation. In the propane purification unit, the propane is dissolved in hexane and separated from CO_2_ and H_2_. Propane is conserved and can be sold as a co-product. CO_2_ and H_2_ are vented or recycled. Propane is either created by breaking the carbon backbone of the triglyceride or formed in the fractionation step. In 2015, the wholesale propane price ranged from $0.4 to $0.8/gal [60].

The liquid products containing all the hydrocarbons are sent to a distillation column. The C_6_–C_8_ hydrocarbons are distillated to the top and the C_9_–C_18_ products are left at the bottom [49, 50, 56] in the second distillation column, where naphtha is purified to the overhead of the column. The naphtha product will be sold as gasoline surrogate. The price of naphtha is $2.0/gal in 2010 US dollars for a 5-year average [24]. The heavier species in the second columns are further separated in the third distillation column. Heavier compounds like C_17_ and C_18_ hydrocarbons that stayed at the bottom are considered diesel alternatives [49, 50]. The overhead stream with hydrocarbons ranging from C_8_ to C_16_ is considered jet fuel range blendstocks. Residual unconverted oil is considered as impurities and a disposal fee would be applied to dispose of the residue stream. Diesel is separated in the fractionation step. The current national average price of biodiesel (B20) is around $2.9/gal and $3.6/gal for biodiesel (B99/B100) [61].

#### Outside battery limits units

All of the wastewater generated in the conversion process is sent to a wastewater treatment (WWT) system, using similar design and cost assumptions as documented in other recent TEA reports [62]. Although this is a costly operation, it yields clean and fully reusable water, which reduces both the fresh makeup water demand and discharge to the environment. All residual oil and unconverted carbon, plus WWT biogas, sludge, and other gas streams, are combusted in an on-site boiler/steam turbine system to produce steam and electricity, which are used to help meet the facility’s energy demands. The costing basis for the boiler/steam turbine and all other utility operations is also maintained consistently with prior recent design cases [62, 63]. The storage area includes storage tanks for propane, hydrocarbon fuels, and water. Water and energy are also integrated for each process.

### Aspen model and techno-economic analysis

The National Renewable Energy Laboratory (NREL) develops and maintains  TEA models that describe the process and production economics of conceptual biochemical conversion pathways to biofuels and bioproducts. For a given set of conversion parameters, material and energy balance and flow rate information are generated using Aspen Plus process simulation software [64], assuming a feed rate to the biorefinery of 788 dry US tons of oil per day. These data are used to size and cost process equipment and compute raw material and other operating costs. Using a discounted cash flow rate of return analysis, the minimum jet fuels selling price (MJSP) required to obtain a net present value of zero for a 10% internal rate of return is determined. The result is a TEA model that reasonably estimates an “*n*th-plant” production cost for this pre-commercial process. Table 2 summarizes the financial assumptions applied in this study.

**Table 2:** *n*th-plant assumptions for TEA [49, 50]

Economic parameters	Assumed basis
Basis year for analysis	2014
Debt/equity for plant financing	60%/40%
Interest rate and term for debt financing	8% annually/10 years
Internal rate of return for equity financing	10%
Total income tax rate	35%
Plant life	30 years
Plant depreciation schedule	7 years
Plant salvage value	0
Construction period	3 years
Fixed capital expenditure schedule	8% in year 1, 60% in year 2 and 32% in year 3
Start-up time	0.5 year
Revenues during startup	50%
Variable costs during startup	75%
Fixed costs during startup	100%
On-stream percentage after startup	90%
Site development costs	9% of ISBL, total installed cost
Warehouse	4% of ISBL
Working capital	5% of fixed capital investment

Indirect costs	% of total direct costs
Prorated expenses	10
Home office and construction fees	20
Field expenses	10
Project contingency	10
Other costs (startup and permitting)	10

Fixed operating costs	Assumed basis
Total salaries	60 employees
Benefits and general overhead	90% of total salaries
Maintenance	3% of ISBL
Insurance and taxes	0.7% of fixed capital investment

*ISBL* inside battery limits (of the plant)

The economic analysis includes a conceptual process design that leads to the development of a detailed process flow diagram (based on research or commercial data); rigorous material and energy balance calculations (via a commercial simulation tool, Aspen Plus); capital and project cost estimations (via an in-house model using spreadsheets); a discounted cash flow economic model; and the calculation of a minimum fuel selling price [62, 65, 66] or MJSP. The operating expense calculation for the designed facility is based on material and energy balance calculations using Aspen Plus process simulations [64]. All costs are adjusted to 2014 US dollars (2014$) using the Plant Cost Index from *Chemical Engineering Magazine* [67], the Industrial Inorganic Chemical Index from SRI Consulting [68], and the labor indices provided by the US Department of Labor Bureau of Labor Statistics [69].

Raw materials include feedstocks (lipid or oil biomass) and chemicals (boiler chemicals, cooling tower chemicals, and makeup amine for the gas cleanup), and upgrading chemicals (catalysts and H_2_) with detailed cost information listed in previous reports and peer-reviewed papers. The feedstock cost varies from $0.40 to $1.75/kg 2014$ depending on the feedstock type shown in Table 3, and the overall process efficiency (or on-stream factor) is assumed to be 90% (7884 operating hours per year), consistent with other TEA design reports [70, 71]. The operating expense calculation for the designed facility is based on material and energy balance calculations using Aspen Plus process simulations [64]. All costs are inflated to 2014$ using the Plant Cost Index from *Chemical Engineering Magazine* [72], the Industrial Inorganic Chemical Index from SRI Consulting [73], and the labor indices provided by the US Department of Labor Bureau of Labor Statistics [74]. Salaries for personnel are inflated to 2014$ [74]. Sixty percent of the total salaries are added for labor burden, and 2.0% of the total installed capital is designated for maintenance (which includes expenses on cleaning) [26]. Property insurance and taxes account for 1.5% of the total capital investment [26]. The federal corporate tax rate used in our analysis is 35% in US. Income tax is averaged over the plant life and that average is calculated on a per-gallon basis. The amount of income tax to be paid by a potential fuel producer varies annually due to changes in the volume of product produced and the allowable depreciation deduction (Additional file 1).

**Table 3:** Oil price [95–103], product yield for a biorefinery with 788 dry ton oil per day

	Jatropha	Camelina	Pennycress	Castor	Yellow grease
Oil price ($/kg)	$0.40	$1.75	$0.81	$1.70	$0.61
Jet fuel production (MMgal/year)	44.0	57.7	40.3	50.8	50.4
Propane fuel yield (gal/dry ton oil)	18.3	1.0	2.1	1.8	1.6
Gasoline fuel yield (gal/dry ton oil)	94.2	100.0	74.3	94.9	93.7
Jet fuel yield (gal/dry ton oil)	170.0	184.3	155.5	196.2	194.7
Diesel yield (gal/dry ton oil)	3.1	4.7	36.9	0.5	0.8

After the total capital investment, variable operating costs, and fixed operating costs are determined, a discounted cash flow rate of return analysis is typically used to determine the minimum fuel selling price (such as MJSP). The discounted cash flow analysis is calculated by iterating the selling cost of the product until the net present value of the project is zero with a 10% internal rate of return. The analysis requires that the discount rate, depreciation method, income tax rates, plant life, and construction start-up duration be specified. The discounted cash flow assumes 40% equity financing with a loan interest at 8% for 10 years. Working capital is assumed to be 5% of the fixed capital investment. The plant is assumed to take 3 years to construct with a half of a year spent on startup. The Internal Revenue Service Modified Accelerated Cost Recovery System (MACRS) was used because it offered the shortest recovery period and largest tax deductions, consistent with several NREL design reports [62, 63, 70, 75], in which the steam production plants depreciates in a 20-year recovery period and all other properties depreciate in a 7-year recovery period. The plant’s life is assumed to be 30 years. The detailed method is described in the previous published NREL design reports [62, 63, 75].

It should be emphasized that our analyses and the resultant MJSP values carry some uncertainty related to the assumptions made about capital and raw material costs. Without a detailed understanding of the basis behind it, the absolute computed cost values have limited relevance. Cost values are therefore best used to compare technological variations or process improvements against one another. By demonstrating the cost impact of various process parameters individually or in concert, the model helps guide research by indicating where the largest opportunities for cost reduction exist.

## Results

### Feedstock analysis

**Fig. 2:**
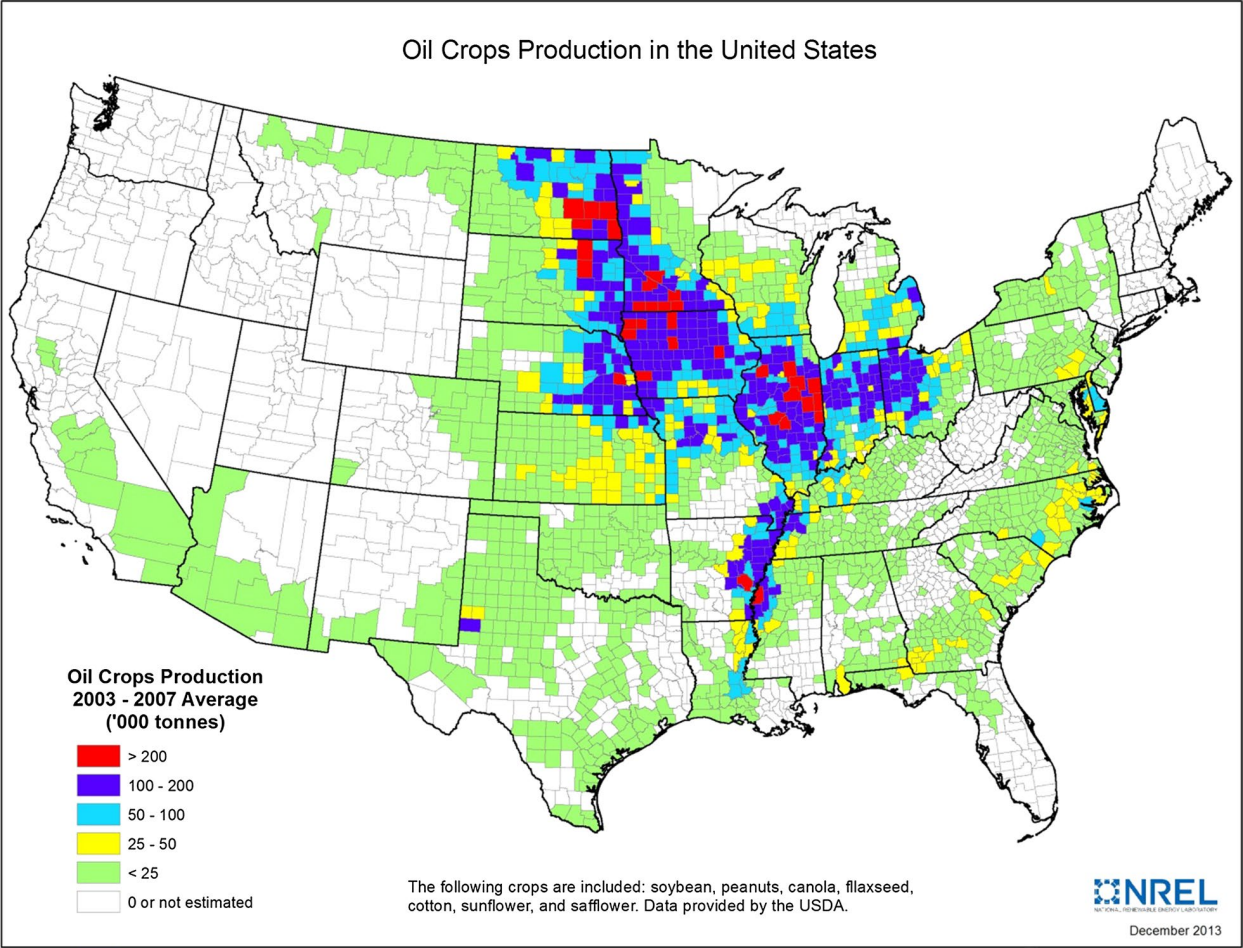
Oil crops production in the US (2003–2007 average)

It is estimated that about 16 million tonnes of vegetable oils, animal fats, and greases are produced annually in the US [76]. About 67% of this amount comes from domestic oil crops, 28% from animal fats and greases, and the remaining from other sources such as tall oil. A variety of oil crops are grown in the US, including soybean, peanut, sunflower, canola, and flax. Production is concentrated in the Corn Belt and along the Mississippi River (Fig. 2). Soybeans are the dominant oilseed in the US, accounting for about 90% of US oilseed production while other oilseeds make up the remainder [77]. The US imports palm, palm kernel, and coconut oil, which are primarily used in the food and chemical industries.

**Fig. 3:**
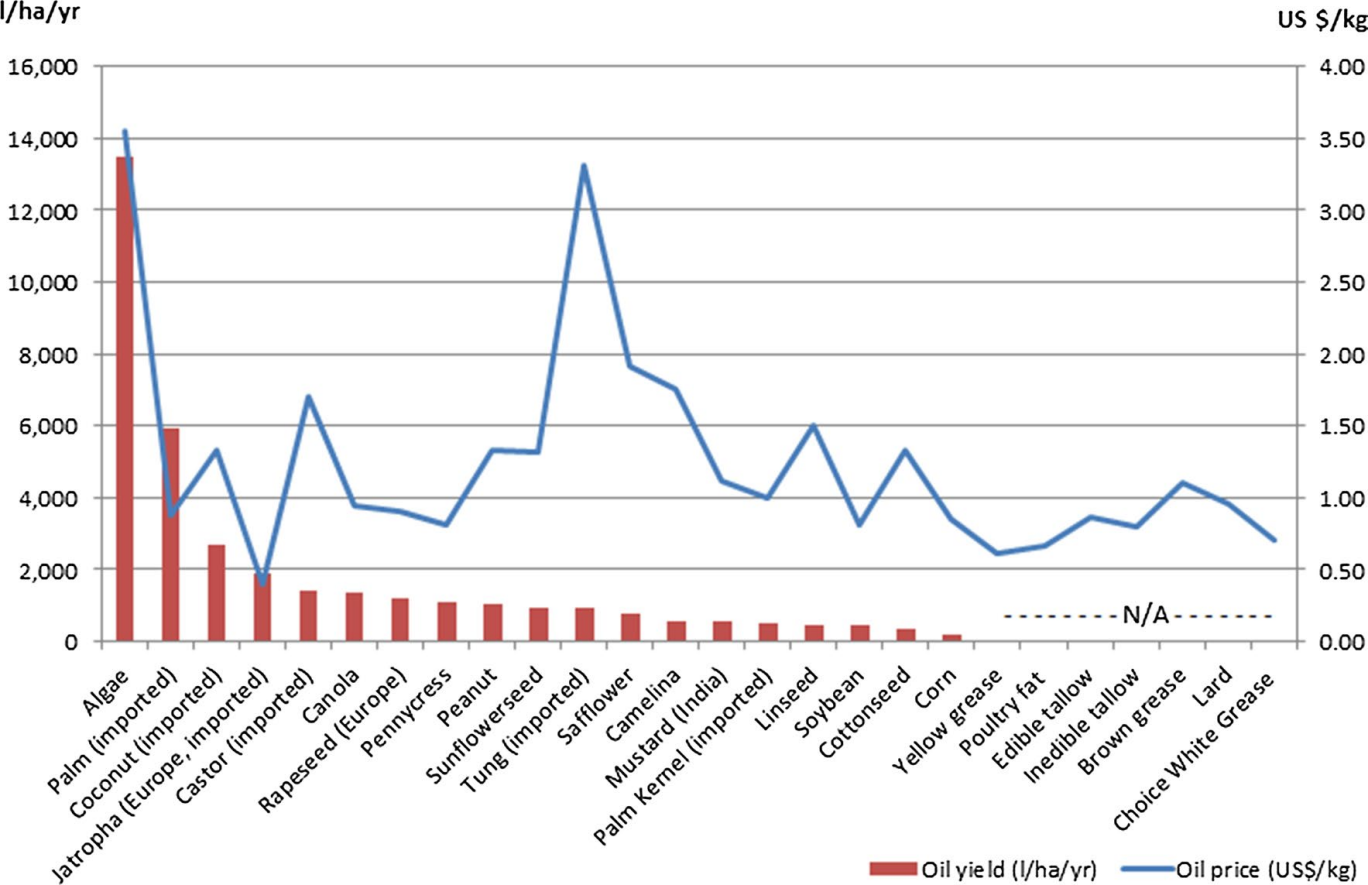
Oil yield and prices. Prices are for local, US feedstock unless otherwise noted. Prices are for 2014, except linseed oil (latest data available from the USDA is for 2010); brown grease (undisclosed time in 2011); safflower and jatropha (2013/2014); mustard (2015); and camelina and algae (model-derived estimates)

Figure 3 illustrates the yield of major oil crops and prices of vegetable oils, animal fats, and greases. Oil crops currently grown in the US (namely soybean) have relatively low oil yield when compared to oil crops grown in other, mainly tropical, parts of the world (e.g., palm, coconut, and jatropha). Algae are expected to have high productivity, which is yet to be proven at commercial scale, but model-derived estimates indicate a prohibitively high price as a biofuel feedstock [29, 78]. Similarly, imported tung oil has a high price and is unlikely to be used as biofuel feedstock.

Castor and pennycress are promising feedstocks for biofuels production given their relatively high yield and because they are non-food oil sources. However, because of its ricinoleic acid content, castor oil is a valuable feedstock for the chemical industry and thus may maintain a higher price than other seed oils even if produced domestically. Castor bean can be grown in the US, as it was in the past and there is revived interest in bringing it back. It, however, would require strong regulations. Canola oil is viewed favorably given its higher-than-soybean yield and is already in use as a biofuels feedstock (for biodiesel production). Lately, however, its use as a biofuels feedstock is facing competition from the food industry, which uses it as a partial replacement for soybean oil and that may lead to prices much higher than other seed oils. Peanut oil also has a higher-than-soybean yield and is more valuable in the market than soybean oil, which makes its use for biofuels production economically impractical. Figure 3 also illustrates that non-terrestrial oil sources such as animal fats and greases have relatively lower prices than terrestrial oil crops. Lower prices and availability has led to increased use of these resources for biofuels production such as biodiesel and renewable diesel in recent years.

### Feedstock fatty acid profile

**Fig. 4:**
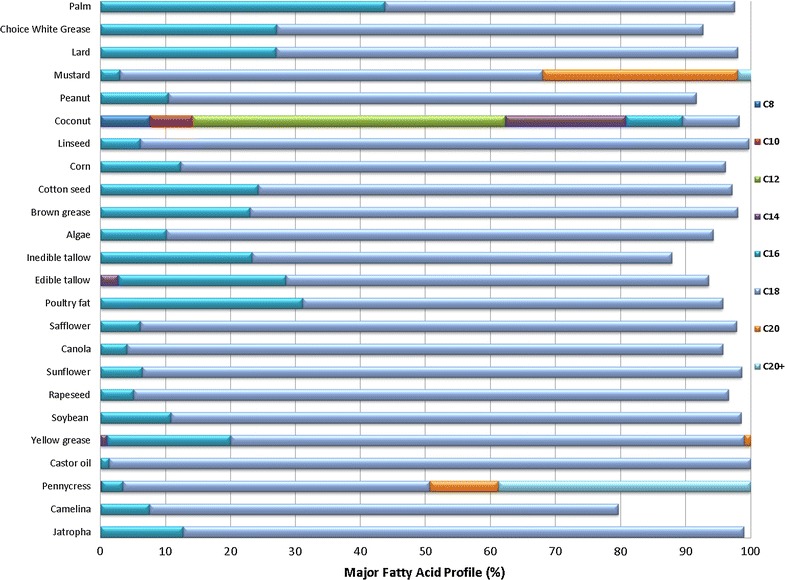
Fatty acid profiles for 24 oil feedstocks

To support our analysis, we collected and analyzed the FFA profile for 24 oil feedstocks. When defining the oil feed, it is assumed that triglycerides, diglyceride and mono-glycerides are main constituents of the bio-oils. For example, in jatropha oil, the compositions of tri-, di-, and mono-glycerides and FFA are 80.4, 2.1, 2.5, and 15.0%, respectively [79]. There are many different types of tri-, di- and mono-glyceride, with the main division between saturated and unsaturated types. The fatty acid compositions are presenting in the form of triglycerides with glycerol in the backbones, also illustrated by Eqs. 4–9. For example, 1 mol triolein is formed by 3 mol of oleic acid. The structure of each of the three fatty acids within a single triglyceride often varies, so the resulted fatty acid profile varies, as listed in Fig. 4 [80–87]. The fatty acids distribute from 8 carbons to 24 carbons. Most oils contain mainly C_16_ and C_18_ FFA. The exceptions are for pennycress, yellow grease, tallow, mustard, and coconut oil.

Oil feedstocks with significant amounts of C_20_ will need hydrocracking (e.g. mustard). Oils with smaller carbon ranges (e.g. coconut oil) would be better candidates for gasoline production. For instance, pennycress has a significant percentage of C_20_. Hydrocracking might be needed for improved jet production. Yellow grease has a small but non-negligible percentage of both C_14_ and C_20_. Hydrocracking will be required for jet production. Wider distribution of carbon numbers would be expected for the resulting hydrocarbon fuels. Edible tallow has a small percentage of C_14_. Mustard has almost 30% of C_20_ and hydrocracking will be required for jet production. Coconut oil has a much wider range of carbons than most other oils with the carbon number ranges from C_8_ to C_16_. The content of C_16_ in coconut oil is only 8%, making it a feedstock candidate for gasoline production, instead of for jet or diesel production.

### TEA results for select feedstocks

**Fig. 5:**
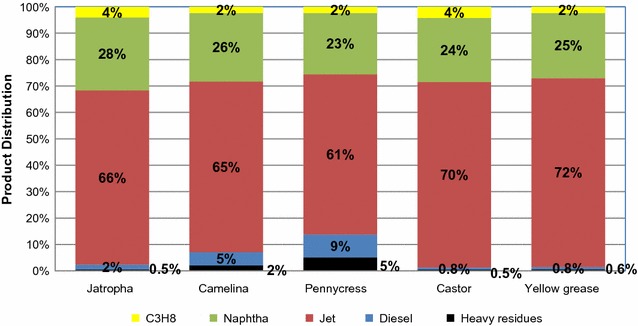
Product distribution of oil-derived hydroprocessed renewable fuel

In jatropha oil, the compositions of tri-, di-, and mono-glycerides and FFA are 80.4, 2.1, 2.5 and 15.0%, respectively [79], with corresponding FFAs shown in Fig. 4. The majority of extracted FFA in jatropha is C_18_. The hydrogenation steps for both saturated and unsaturated triglycerides are critical for upgrading jatropha oil, due to the high content of triglycerides. The high triglycerides content also results in a high yield of propane, as illustrated in Fig. 5. The resulting FFAs, however, are mostly in the range of C_8_–C_18_, so hydrocracking mainly cracks C_15_ and C_17_. The final product and co-products, including jet, diesel, naphtha, and propane, are illustrated in Fig. 5. The HEFA using jatropha oil produces 32% naphtha, 62% jet, 1% diesel, and 5% propane. With feedstock throughput of 788 dry tons oil per day, the production rate of each product and co-product are summarized in Table 3. Hydrocracking is applied whenever possible to maximize jet hydrocarbon productions.

Camelina has a typical oil content of 40% and can produce higher amounts of α-linolenic acid. Camelina (false flax) oil is an important source of linolenic acid (C_18:3_) [88]. We have assumed 100% FFA for camelina oil in the TEA, so the first hydrogenation step is almost bypassed with low production of propane. Similar to jatropha, the FFAs are mostly in the range of C_8_–C_18_, so hydrocracking mainly cracks C_15_ and C_17_. Production yields are summarized in Table 3.

The oil content of dried field pennycress seeds is 29.0 wt%. The primary FFA in pennycress is erucic acid (32.8 wt% of C_22:1_), which is typical among members of the Brassicaceae family [34]. With significant amounts of C_20_ and C_22_ in the pennycress oil, the hydrocracking mainly cracks C_15+_. Because pennycress has a significant percentage of C_20+_, even with a hydrocracker, the diesel yield (shown in Fig. 5 and Table 3), is still significantly higher than that from the other oils. Malaysian castor seeds contain a relatively high percentage of oil, and total lipids content is 43.3% (per dry weight) [89]. The unsaturated fatty acids content was 97.5% of the total fatty acids composition. Oil feedstocks with unsaturated fatty acid contents typically require higher amount H_2_ to remove the OH groups. Ricinoleic acid comprises over 84% while other fatty acids present are linoleic (7.3%), oleic (5.5%), palmitic (1.3%), stearic (1.2%), and linolenic (0.5%) [89] (Fig. 4). Similar to jatropha, the FFAs are mostly in the range of C_8_–C_18_, so hydrocracking mainly cracks C_15_ and C_17_.

Lower cost feedstocks such as animal fats, yellow grease, and brown grease are high in FFA [90], with the range of C_8_–C_18_. Although yellow grease has a small but non-negligible percentage of both C_14_ and C_20_ and wider distribution of carbon numbers, the jet blendstock yield is comparable with other oil feedstocks, such as jatropha, camelina, and castor oil, indicating a great potential of using the low-grade oil as a good feedstock candidate for making hydrocarbon fuels via oil upgrading.

**Fig. 6:**
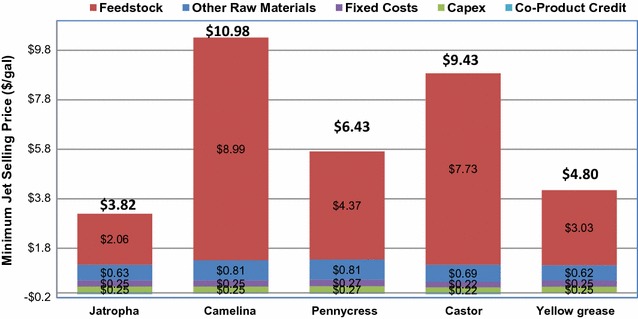
MJSP for five oil feedstocks

If the oil feedstock is predominately a C_16_–C_18_ oil, the products are mostly diesel fuel range molecules without the hydrocracking step. Thus, with the addition of the hydrocracking step more jet fuel is produced by catalytically cracking diesel range molecules. The product profile is illustrated in Fig. 5, showing results of the distribution of propane, naphtha, jet, diesel and heave residuals from the five selected oil feedstocks after catalytic oil upgrading and fractionation unit operations. In addition, Table 3 shows the mass-based product yields. In summary, jet fuel ranges from 60 to 70% for the selected five oil feedstocks. When compared with the data from literature [24], the yields of propane and naphtha are similar. Propane accounts for 2–4% in weight of all the products, strongly correlated with the tri-, di- and mono-glycerides contents in the oil feedstocks. In our case, more hydrocarbons are distributed in the jet fuel pool because cracking reactions are assumed in the hydrocracker. Moreover, more CO_2_ is presented because only decarboxylation is represented for the deoxygenation process if compared with that in the study done by Pearlson et al. [24] in which both decarboxylation and hydrodeoxygenation are assumed. Product yields and distribution are generally consistent with data from the published TEA using soybean oil as the feedstock [24]. The estimated MJSP is shown in Fig. 6, including feedstock, other operating cost (OPEX) and capital contributions.

In this study, both camelina and castor bean prices are high, resulting in over 80% cost contribution from feedstock costs (see Table 3). The feedstock contribution for the other oils range from 55 to 69%. Similar to the literature, 76–88% of the total production cost is contributed by the cost of feedstocks [91–94]. Capital investment is similar for all five processes with selected feedstocks, ranging from $341 to $354 million for total capital investment and contributing 10–25% of overall jet production cost. Total capital cost includes the capital depreciation and return on capital. Cost contribution from other OPEX has H_2_ consumption in the oil upgrading steps, catalysts costs, and additional utility costs. Utilities must be purchased for the HEFA facilities unless there is an on-site boiler and combined heat and power. The MJSPs shown in Fig. 6 are calculated based on jet blendstocks as the main products, while selling propane, diesel, and gasoline blendstocks as co-products. The MJSP ranges from $3.8 to $11.0/gal jet. The big variations of MJSP for the selected five oil feedstocks are mainly due to differences in oil prices. Variations on capital costs are relatively small.

**Fig. 7:**
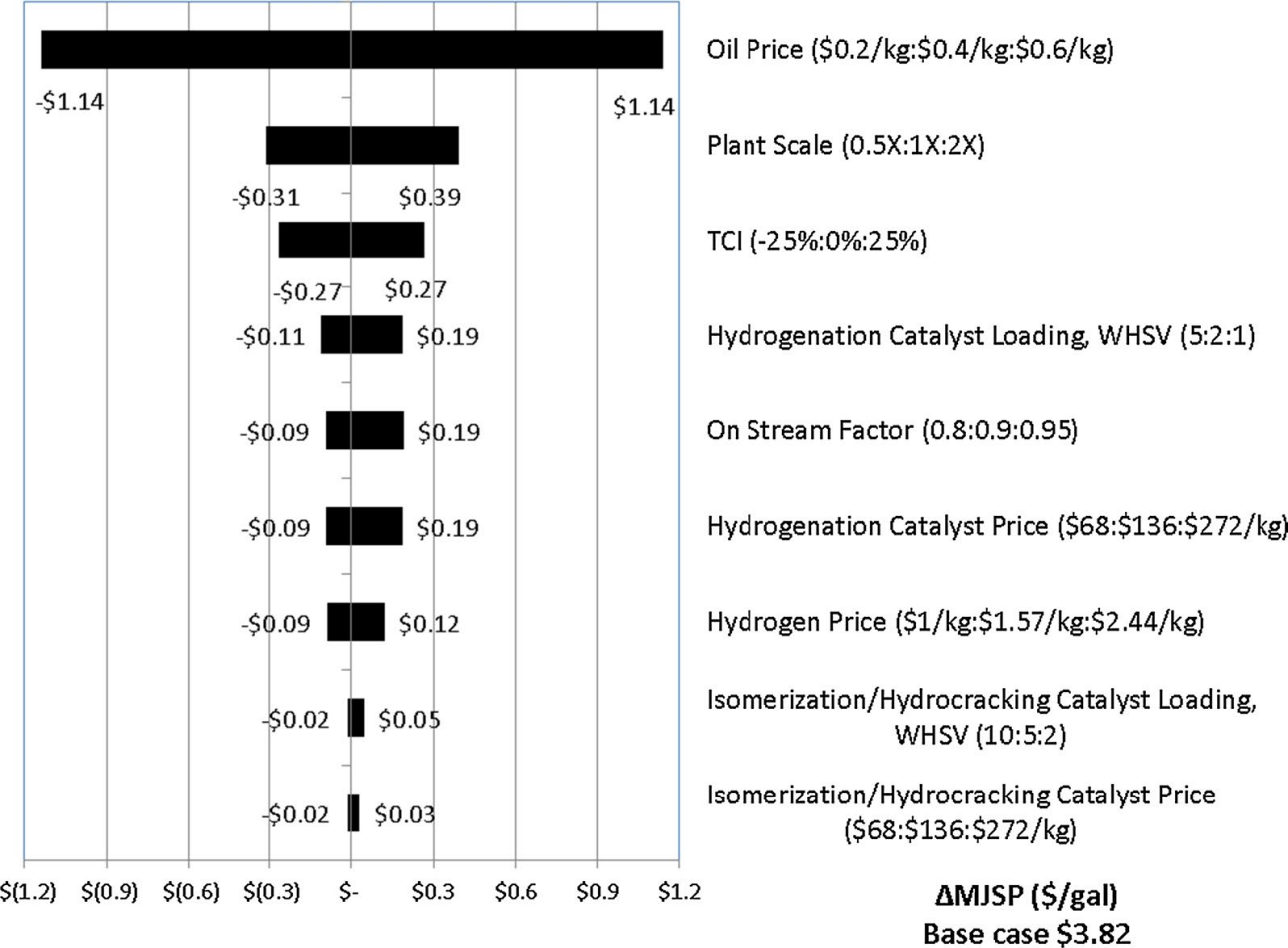
Single point sensitivity for MJSP of jatropha oil

A single-point sensitivity analysis is performed on the HEFA process using jatropha oil. Minima and maxima for each variable are chosen to understand and quantify the resulting cost impact on overall MJSP. Each variable is changed to its minimum and maximum value with all other factors held constant. Most correlations are linear, except the correlation between plant scale and MJSP. The results and limits are shown in Fig. 7. The oil price, plant capacity, total capital investment, oil upgrading catalyst loadings, process efficiency and catalyst prices, and total capital investment have the largest impact on MJSP. Therefore, they are key cost drivers. The feedstock (oil) price, catalysts loadings and prices, and H_2_ price are positively correlated to MJSP. Plant scale, process efficiency and jet fuel yields also have a strong impact on MJSP, but they are negatively correlated. The other parameters chosen for this study (such as isomerization and hydrocracking catalyst price) show minimal contribution to MJSP. It is noted that pathways from different oil feedstocks follow similar patterns for this sensitivity study. Beside the other variables mentioned as the largest cost drivers, new developments in reactor type (for hydrotreating, propane cleave, or for hydrocracking and hydroisomerization) could reduce the MJSP significantly.

## Conclusions

The resource analysis indicates that oil crops currently grown in the US (such as soybean) have relatively low oil yield when compared to oil crops grown in other, mainly tropical, parts of the world (e.g., palm, coconut, and jatropha). Higher-yielding oil crops such as canola and camelina are increasingly grown in the country but they are facing competition with the food industry; thus it is unclear what the future holds for these resources. While receiving a lot of attention, pennycress and jatropha are slow to develop for various reasons (e.g., agronomic, economic, and social). Non-terrestrial oil sources such as animal fats and greases have relatively lower prices than terrestrial oil crops and thus are increasingly used for biofuels production. With inputs from resource analysis on feedstock compositions profiles, oil prices, and availability, TEA is performed for five selected oil feedstocks using the HEFA process concept. The five selected oils are camelina, pennycress, jatropha, castor bean, and yellow grease. Please note that there are no mature feedstock markets at the moment available for the four oilseeds analyzed, and the feedstock prices are still quite volatile in the current market. For instance, the MJSP for these five resources ranges between $3.8 and $11.0 per gallon jet blendstocks, mainly due the variation of oil feedstock prices. If feedstock price can be assumed the same, the MJSP variation is small. Feedstock is the main component of MJSP for HEFA. Jet fuel generally comprises around 60% of output for the oil feedstocks studied in this work. Sensitivity analysis indicates that the key cost drivers are feedstock price, conversion plant capacity, fatty acid profile, addition of hydrocracker, and type of hydroprocessing catalysts. Both edible and non-edible oils are promising alternative fuel feedstocks not only because they are renewable and can be produced locally and in environmentally friendly ways, but also because they can be cost competitive with strategic process design and integration, taking into consideration oil prices, resources, and feedstock composition profiles. Because there are currently no mature feedstock markets available for the four oilseeds analyzed, uncertainty analysis will be conducted in the future.

## Additional file

**Additional file 1.** Additional Explanation on the Terminologies.

## Abbreviations

AJF: alternative jet fuel
HEFA: hydroprocessed esters and fatty acids
ASTM: American Society for Testing and Materials
EIA: Energy Information Administration
FFA: free fatty acid
GHG: greenhouse gas
HFA: hydroxylated fatty acids
HRJ: hydroprocessed renewable jet
ISBL: inside battery limits (of the plant)
LPG: liquefied petroleum gas
MJSP: minimum jet fuel selling price
TEA: techno-economic analysis
SPK: synthetic paraffinic kerosene
USDA: US Department of Agriculture
WWT: wastewater treatment
