# Supplementary material for: Techno-economic and resource analysis of hydroprocessed renewable jet fuel
Source: Biotechnol Biofuels. 2017 Nov 9;10:261. doi: 10.1186/s13068-017-0945-3 (PMC5679388; doi:10.1186/s13068-017-0945-3)
Supplement: Supplementary file 1 — Additional file 1. Additional Explanation on the Terminologies. [file 13068_2017_945_MOESM1_ESM.docx]

## Additional file

Working Capital. Peters and Timmerhaus [[1](#_ENREF_1), [2](#_ENREF_2)] define working capital as money available to cover (1) raw materials and supplies in inventory, (2) finished product in storage, (3) accounts receivable, (4) cash on hand for monthly payments such as wages and maintenance supplies, (5) accounts payable, and (6) taxes payable. This flow of money is required over the life of the plant, beginning in the start-up phase to make product that generates revenue to use in purchasing more materials and supplies. Peters and Timmerhaus [[1](#_ENREF_1), [2](#_ENREF_2)] indicate that working capital is usually 5%-20% of the total capital investment. In order to be consistent with other NREL analysis, as well as in previous NREL design reports [[3-5](#_ENREF_3)], we use a consistent working capital of 5% of FCI.

The Modified Accelerated Cost Recovery System (MACRS) is used to recover the basis of most business and investment property placed in service after 1986. MACRS consists of two depreciation systems, the General Depreciation System (GDS) and the Alternative Depreciation System (ADS). Generally, these systems provide different methods and recovery periods to use in figuring depreciation deductions. We applied a consistent MARCS method with GDS, recommended by Short [[6](#_ENREF_6)] to determine the depreciation amount for the calculation of federal taxes to be paid. This GDS allows both the 200% and 150% declining balance (DB) methods of depreciation. This offers the shortest recovery period and the largest tax deductions. IRS publication 534 [[7](#_ENREF_7)] indicates that steam production plants should use a 20-year recovery period. The IRS indicates that other property not specifically described in the publication should be depreciated using a 7-year recovery period. The MACRS depreciation method is consistent with all NREL design reports [[3-5](#_ENREF_3)], which have more detailed information.

Inflation rate. The operating expense calculation for the designed facility is based on material and energy balance calculations using Aspen Plus process simulations [[8](#_ENREF_8)]. All costs are inflated to 2014 U.S. dollars using the Plant Cost Index from Chemical Engineering Magazine[[9](#_ENREF_9)], the Industrial Inorganic Chemical Index from SRI Consulting [[10](#_ENREF_10)], and the labor indices provided by the U.S. Department of Labor Bureau of Labor Statistics[[11](#_ENREF_11)]. Salaries for personnel are inflated to 2014 dollars[[11](#_ENREF_11)]. Sixty percent of the total salaries are added for labor burden, and 2.0% of the total installed capital (TIC) is designated for maintenance (which includes expenses on cleaning) [[12](#_ENREF_12)]. Property insurance and taxes account for 1.5% of the total capital investment (TCI)[[12](#_ENREF_12)]. The federal corporate tax rate used in our analysis is 35%. Income tax is averaged over the plant life and that average is calculated on a per-gallon basis. The amount of income tax to be paid by a potential fuel producer varies annually due to changes in the volume of product produced and the allowable depreciation deduction.

Definition of nth-Plant Economics [[3-5](#_ENREF_3), [13](#_ENREF_13)]. The key assumption associated with nth-plant economics is that several plants using the same technology have already been built and are operating. In other words, the assumption reflects a future in which a successful industry has been established with many operating plants. Because the techno-economic model is a tool used primarily for (1) studying new process technologies or (2) comparing integrated processes in order to comment on their relative economic impact, it is prudent to ignore artificial inflation of project costs associated with risk financing, longer start- ups, equipment overdesign, and other costs associated with pioneer plants, as these overshadow the real economic impact of advances in conversion science or process engineering research. At the very least, nth-plant economics should help to provide justification and support for early technology adopters and pioneer plants about longer-term prospects. Because equipment costs in this design report have been estimated explicitly, the nth-plant assumptions apply primarily to the factored cost model used to determine the total capital investment from the purchased equipment cost and to the assumptions applied for plant financing. The nth-plant assumption also applies to operating parameters, such as process uptime and start-up time. A summary of the nth-plant assumptions applied in are listed in Table 2. These financial assumptions are consistent with assumptions used for other economic analyses done for DOE’s Bioenergy Technologies Office, with some deviations to reflect the uniqueness of the current processes. The key assumption implied by nth-plant economics is that our analysis does not describe a pioneer plant; instead, it assumes several plants using the same technology have already been built and are operating. In other words, it reflects a mature future in which a successful industry of n plants has been established. Because the techno-economic model is primarily a tool for studying new process technologies or integration schemes in order to comment on their comparative economic impact, nth-plant analysis avoids artificial inflation of project costs associated with risk financing, longer startups, equipment overdesign, and other costs associated with first-of-a-kind or pioneer plants, lest these overshadow the economic impact of research advances in conversion or process integration . At the very least, these nth-plant economics should help to provide justification and support for early technology adopters and pioneer plants. We will work on quantifying economic factors associated with first-of-a-kind implementation as our next steps.

### Reference:

1. Peters M, Timmerhaus K: *Plant Design and Economics for Chemical Engineers.* 4th Edition edn. New York City: McGraw-Hill; 1991.

2. Peters MS, Timmerhaus KD, West RE, Timmerhaus K, West R: *Plant design and economics for chemical engineers.* McGraw-Hill New York; 2003.

3. Aden A, Ruth M, Ibsen K, Jechura J, Neeves K, Sheehan J, Wallace B, Montague L, Slayton A, Lukas J: **Lignocellulosic Biomass to Ethanol Process Design and Economics Utilizing Co-Current Dilute Acid Prehydrolysis and Enzymatic Hydrolysis for Corn Stover.** In *Other Information: PBD: 1 Jun 2002*. pp. Medium: ED; Size: 154 pages; 2002:Medium: ED; Size: 154 pages.

4. Davis R, Tao L, Tan EC, Biddy MJ, Beckham G, Scarlata CJ, Jacobson J, Cafferty K, Ross J, Lukas J, et al: **Process Design and Economics for the Conversion of Lignocellulosic Biomass to Hydrocarbons-Dilute-Acid and Enzymatic Deconstruction of Biomass to Sugars and Biological Conversion of Sugars to Hydrocarbons.** 2013.

5. Humbird D, Davis R, Tao L, Kinchin C, Hsu D, Aden A, Schoen P, Lukas J, Olthof B, Worley M, et al: **Process Design and Economics for Biochemical Conversion of Lignocellulosic Biomass to Ethanol: Dilute-Acid Pretreatment and Enzymatic Hydrolysis of Corn Stover.** 2011.

6. Short W, Packey DJ, Holt T: **A Manual for the Economic Evaluation and Energy Efficiency and Renewable Energy Technologies.** Golden CO: National Renewable Energy Laboratory; 1995.

7. Service IR: **How to Depreciate Property.** (Treasury Dot ed. Washington, D.C.; 2009.

8. AspenPlus^TM^: **Release 7.2, Aspen Technology Inc., Cambridge MA.** 2007.

9. **Chemical Engineering Magazine** [<http://www.chemengonline.com/pci-home>]

10. SRI Consulting: **U.S. Producer Price Indexes – Chemicals and Allied Products/Industrial Inorganic Chemicals Index.** In *Chemical Economics Handbook.* 2008

11. **Bureau of Labor Statistics Data website, National employment, hours, and earnings catalog, industry: chemicals and allied products, 1980-2009.** [<http://data.bls.gov/cgi-bin/srgate>]

12. Davis R, Fishman D, Frank E, Wigmosta M, Aden A, Coleman A, Pienkos P, Skaggs R, Venteris E, Wang M: **Renewable diesel from algal lipids: an integrated baseline for cost, emissions, and resource potential from a harmonized model.** Golden, CO: National Renewable Energy Laboratory (NREL); 2012.

13. Dutta A, Sahir A, Tan E, Humbird D, Snowden-Swan LJ, Meyer P, Ross J, Sexton D, Yap R, Lukas J: **Process Design and Economics for the Conversion of Lignocellulosic Biomass to Hydrocarbon Fuels Thermochemical Research Pathways with In Situ and Ex Situ Upgrading of Fast Pyrolysis Vapors.** National Renewable Energy Laboratory: National Renewable Energy Laboratory; 2015.
